# Microbial-transcriptome integrative analysis of heat stress effects on amino acid metabolism and lipid peroxidation in poultry jejunum

**DOI:** 10.1080/10495398.2024.2331179

**Published:** 2024-03-22

**Authors:** Young-Jun Seo, Chiwoong Lim, Byeonghwi Lim, Jun-Mo Kim

**Affiliations:** Department of Animal Science and Technology, Chung-Ang University, Anseong, Republic of Korea

**Keywords:** Heat stress, broiler, microbiome, RNA-seq, multi-omics integration

## Abstract

Despite the significant threat of heat stress to livestock animals, only a few studies have considered the potential relationship between broiler chickens and their microbiota. Therefore, this study examined microbial modifications, transcriptional changes and host–microbiome interactions using a predicted metabolome data-based approach to understand the impact of heat stress on poultry. After the analysis, the host functional enrichment analysis revealed that pathways related to lipid and protein metabolism were elevated under heat stress conditions. In contrast, pathways related to the cell cycle were suppressed under normal environmental temperatures. In line with the transcriptome analysis, the microbial analysis results indicate that taxonomic changes affect lipid degradation. Heat stress engendered statistically significant difference in the abundance of 11 microorganisms, including *Bacteroides* and *Peptostreptococcacea*. Together, integrative approach analysis suggests that microbiota-induced metabolites affect host fatty acid peroxidation metabolism, which is correlated with the gene families of Acyl-CoA dehydrogenase long chain (*ACADL*), Acyl-CoA Oxidase (*ACOX*) and Acetyl-CoA Acyltransferase (*ACAA*). This integrated approach provides novel insights into heat stress problems and identifies potential biomarkers associated with heat stress.

## Introduction

Elevated environmental temperatures severely affect livestock,^1^^,^^2^ with poultry being extensively susceptible to ambient conditions.^3^ Heat stress is the foremost lethal stressor in chickens, attenuating feed intake, growth rates, meat productivity and quality.^4–6^ Previous studies have evidenced that heightened environmental conditions amplify reactive oxygen production and inflammation reactions,^7^^,^^8^ prompting intestinal impairments and epithelial barrier dysfunction.^6^^,^^9^ Vital functions in chicken intestines afflicted by heat stress include nutrient digestion, absorption and pathogen protection.^10^^,^^11^ Specifically, the immune and microbial barrier in the jejunum, the central section of the small intestine, plays a crucial role in maintaining gut health in broiler chickens.^12^ Thus, additional molecular and cellular mechanistic investigations centred on jejunal transcriptome profiling will provide further insight into gene expression changes, complex genomic heat stress responses and genetic heat tolerance regulation in chickens.^13^^,^^14^ Furthermore, alterations in gut microorganisms as a result of heat stress have recently garnered attention as a significant factor in explaining heat stress.^15^^,^^16^ It has been confirmed that heat stress leads to a significant change in the intestinal environment, causing an imbalance in intestinal microorganisms resulting in phenotypic changes in the host.^17^^,^^18^ Previous studies have attempted to determine the effects of heat stress on jejunal tissue and microbiota using a single-omic approach; however, this method cannot establish molecular change and gene expression relationships in specific circumstances.^19^^,^^20^

The biological system of animals comprises complex regulatory features and is sensitive to environmental factors that influence host phenotypes and gut function.^21^ In addition to regulatory heat stress responses, the microbiome can fluctuate host gene and metabolite expressions. Thus, each component and its interactions must be investigated to understand heat stress mechanisms.^22^^,^^23^ In recent observations, the microbiome directly affected stress-associated hormones and mediators in bacterial pathogens.^24^ Although previous research has elucidated the significance of microbiome through chicken microbiome analyses, few have compared functional changes predicated on heat stress.

Microbiome and transcriptome integration approaches enable comprehensive insights into heat stress mechanisms by predicting microbiota-derived metabolites, unveiling how the microbiome mitigates or enhances heat stress conditions.^25^^,^^26^ These approaches are expected to present continuous and direct microfloral strategies for managing continuously rising environmental temperatures and various heat stress concerns. This study employs integrative approaches to analyse transcriptional and microbial changes in the broiler intestine in response to heat stress. Thus, we focused on gaining a comprehensive understanding of heat stress responses and identifying potential microbiota and genes for reducing economic losses by mitigating phenotypic changes.

## Materials and methods

### Experimental animals and sample collection

The Institutional Animal Care and Use Committee (IACUC; No. 2020-00022) approved this study. We utilized animal samples described by Kim et al. for broilers in our study.^27^ A total of 170 male broiler chicks from the Ross 308 strain were acquired at 1 day of age from a local hatchery (Dongsan broiler hatchery, situated in Cheonan-si, Korea.) Before the commencement of the experiment, all these chicks were provided with a commercial diet to meet their energy and nutritional requirements. When the broiler chickens reached 21 days of age, their weights were recorded, and 50 chickens with significantly high or low body weights were excluded from the study, leaving us with 120 broiler chickens. These remaining broilers had an average body weight of 866 ± 61.9 g. Subsequently, the broilers were divided into two treatment groups, each further allocated to six separate cages. This allocation was carried out using a completely randomized design.

The thermoneutral group of broilers was reared in a controlled environment with a temperature of 20 °C and a relative humidity of 57% throughout the entire experimental period. In contrast, heat-stressed group of broilers was subjected to a cyclic heat stress circumstance, experiencing temperatures ranging from 31 to 32 °C for 8 h a day and 27 to 28 °C for the rest of the time. Both groups of Broilers were provided with a basal diet which was carefully formulated to meet or surpass the nutritional requirements specified in the Ross 308 manual^28^ and 23-h lighting program was used in the experiment. We selected the three broilers that have growth performance close to average in each group for bioinformatic analysis. The animals were euthanized on Day 42; jejunal mucosa and contents were collected for mRNA and 16S rRNA extractions. A sterile slide scraped jejunal mucosa from the inner intestinal tissue. All samples were collected in sterilized tubes, frozen using liquid nitrogen and stored at −80 °C.

### RNA extraction, library preparation and sequencing

A 1 ml TRIzol reagent (Invitrogen, Carlsbad, CA, USA) extracted 50 mg of six jejunal sample’s total RNA, which the NanoDrop (Thermo Scientific, Waltham, MA, USA) assessed for quality. The samples that are RNA integrity number (RIN) over the 7 were used in further analysis. A cDNA library was independently generated for each sample using the Illumina TruSeq Stranded mRNA Sample Prep Kit (Illumina, Inc., San Diego, CA, USA) and 1 µg of total RNA. The initial step involved eliminating rRNA from the total RNA using the Ribo-Zero rRNA Removal Kit (Human/Mouse/Rat; Illumina, Inc.). Following this, the remaining mRNA underwent fragmentation into small pieces through exposure to divalent cations at elevated temperatures. The resulting cleaved RNA fragments were then reverse-transcribed into first-strand cDNA using SuperScript II reverse transcriptase (Invitrogen, Life Technologies) and random primers. Subsequently, second-strand cDNA synthesis was performed using DNA Polymerase I, RNase H and dUTPs. The cDNA fragments underwent an end-repair process, including the addition of a single ‘A’ base, followed by the ligation of adapters. The resulting products were purified and enriched through PCR to yield the final cDNA library. Quantification of the libraries was carried out using KAPA Library Quantification kits for Illumina Sequencing platforms, following the qPCR Quantification Protocol Guide (Roche, Basel, Switzerland). Validation of the libraries was conducted using the TapeStation D1000 ScreenTape System (Agilent Technologies, Santa Clara, CA, USA). Messenger RNA sequencing was achieved using an Illumina HiSeq 4000 (Illumina Inc., San Diego, CA, USA) sequencer with paired-end, 100 base-pair reads.

### Differentially expressed gene profiling

Raw read data quality was assessed using FastQC,^29^ and low-quality adapter sequences and reads were removed using Trimmomatic v.0.39 to separate clean reads for further analysis.^30^ The Gallus_gallus (GRCg6a, GCA_000002315.5) reference genome in the FASTA format was obtained from Ensemble (http://www.ensembl.org/). Clean, paired-end reads were mapped against an indexed reference genome using HISAT2,^31^ a sensitive and swift alignment program for next-generation sequencing reads. Raw counts corresponding to each library’s genes were calculated based on exons; the Gallus_gallus v101 GTF file was implemented as the genomic annotation reference file using FeatureCounts’ R package ‘Subread’ function.^32^

Raw counts were normalized through the trimmed means of *M* values (TMM) method in the ‘edgeR’ package,^33^ followed by DEG profiling from normalized read count comparisons between the heat-stressed and thermoneutral groups. The false discovery rate (FDR) was calculated using the Benjamini–Hochberg procedure. Significant DEGs were extracted by applying FDR < 0.05 and absolute log_2_ fold change (FC) ≥ 1 thresholds; overall gene expressions were visualized using the ‘ggplotR’ package.

### Functional enrichment analyses

The Database for Annotation, Visualization and Integrated Discovery (DAVID) bioinformatics resources 6.8 were employed to examine the Kyoto Encyclopedia of Genes and Genomes (KEGG) and gene ontology (GO) databases for functional enrichment analyses of DEGs; KEGG and GO significance in each stage were detected at *p* < 0.1, and results were visualized by using − log_10_* p* and log_2_ fold enrichment as criteria. GO enrichment analysis assessed biological process, cellular component and molecular function. REVIGO visualized these enriched GO terms as treemaps.^34^

Expression data for the gene set enrichment analysis (GSEA) were collated using TMM-normalized count data; all gene expression differences were ranked to ascertain patterns between groups. The GO database processed the gene set data, parameters were set at their default values, and gene sets were ranked by calculating normalized enrichment scores (NESs). Cytoscape (v.3.7.2) visualized the results as a functional enrichment map.^35^ The cut-off value was FDR < 0.01; the similarity cut-off was a >0.8 overlap coefficient.

### Microbial DNA extraction, library construction and sequencing

Sequencing libraries were prepared following Illumina 16S Metagenomic Sequencing Library protocols to amplify the V3 and V4 regions using 3 ml of jejunal contents in each 6 samples. The input gDNA 2 ng was PCR-amplified with a 5× reaction buffer, 1 mM of dNTP mix and 500 nM of the universal F/R PCR primer and Herculase II fusion DNA polymerase (Agilent Technologies, Santa Clara, CA). Initial PCR cycle conditions were 3 min at 95 °C for heat activation and 25 cycles of 30 s at 95 °C, 30 s at 55 °C and 30 s at 72 °C, followed by a final 5-min extension at 72 °C. The universal primer pair with Illumina adapter overhang sequences for the first amplification was as follows: V3-F: 5′-TCGTCGGCAGCGTCAGATGTGTATAAGAGACAGCCTACGGGNGGCWGCAG-3′, V4-R: 5′- GTCTCGTGGGCTCGGAGATGTGTATAAGAGACAGGACTACHVGGGTATCTAATCC-3′. The first PCR product was purified with AMPure beads (Agencourt Bioscience, Beverly, MA), and then 2 μl was PCR-amplified using the Nextera XT kit (Illumina, San Diego, CA) for final library construction. The second PCR cycle conditions matched the first, except for 10 cycles. The PCR product was purified with AMPure beads, quantified using qPCR adhering to the qPCR Quantification Protocol Guide (KAPA Library Quantification kits for Illumina Sequencing platforms) and qualified using the TapeStation D1000 ScreenTape (Agilent Technologies, Waldbronn, Germany). Paired-end (2 × 300bp) sequencing was achieved by the MiSeq™ platform (Illumina, San Diego, USA).

### Microbiome dataset processing and diversity analysis

Cutadapt v3.7 trimmed trivial 16S rRNA sequence adaptors for further study, and QIIME2 v2020.03 processed all 16S rRNA gene sequences. Concerning the microbiome dataset, noisy and amplicon sequence errors were filtered (minimum length ≥ 5, quality score ≥ 25) while the DADA2 algorithm generated amplicon sequence variants (ASVs). A Naïve Bayes classifier (SILVA 132) trained for 16S rRNA V3–V4 hypervariable region using the q2-feature-classifier plugin assigned representative sequences taxonomy. ASVs, resulted from DADA2, were classified against the trained SILVA v132 reference database. Identified taxa underwent differential abundance analysis with a linear discriminant analysis effect size (LEfSe) of >2.0 LDA.

The Qiime2 core-metrics-phylogenetic and alpha-group-significance script estimated richness (observed ASVs) and Shannon Diversity Indices for the alpha-diversity analysis.^36^ Similarly, beta diversity was assessed via non-metric multidimensional scaling ordination from ASV Bray–Curtis dissimilarity in the Qiime2 core-metrics-phylogenetic and alpha-group-significance script.

### Microbiota functional annotation

Phylogenetic Investigation of Communities by Reconstruction of Unobserved States (PICRUSt2) predicted the functional gut microbiome from 16S rRNA genes in the Silva database gene sequences’ phylogenetic tree. Representative sequence data was input for metagenome prediction rarefied, and the ASV table used each sample’s 16S rRNA gene count for normalization. To improve accuracy of gene prediction and reduce the fluctuations in microbiota, there is copy number normalization process after gene content prediction by PICRUSt2. Composition profiles were annotated into the KEGG pathway database. Each pathway’s significance was calculated through t-tests and identified as significant at a *p* value <0.05. STAMP v2.1.3 visualized identified pathways.^37^

### Host–microbiota interaction analysis

The PICRUSt2 database used KEGG Orthology (KO) as a query to identify microorganisms associated with the selected significant pathways. We compiled a presence/absence list of compounds contributing to these significant pathways from the KEGG pathway database and excluded metabolites without InChIKey from further analysis. Hostmetabolic contribution was determined using the STITCHv5.0 database.^38^ STITCH offers a valuable resource for searching through established as well as potential interactions between chemicals and proteins.^38^ This is made possible through the integration of information from diverse sources, such as metabolic pathways, crystal structures, binding experiments and drug-target relationships.^38^ Interactions with high confidence scores (>0.9) and known functional effects were identified, and a PPI network analysis for each predicted metabolite set was conducted.

Overlapped transcriptome and integration analysis pathways were further analysed as described by MacMillan et al.^39^ Contributing taxa and host transcriptome genes were selected and examined through Pathview to distinguish potent gene and protein modulations.^40^ Analysis with the Spearman correlation for overlapped pathways was performed on host genes and taxa, including pathway-contributing DEGs and microbiota associated with more than four KOs in the pathway and present in more than half of all samples.

## Results

### RNA-seq data and DEG profiling

**Figure 1.**
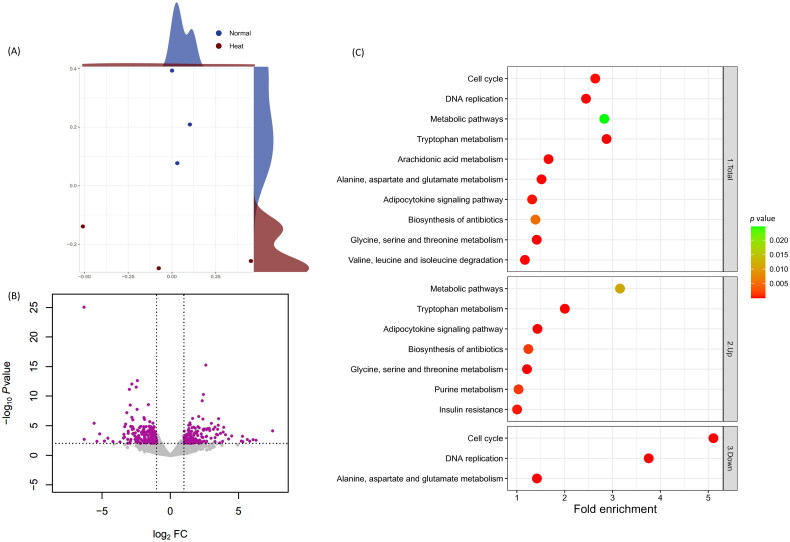
RNA-seq transcriptional profiling in the chicken cecum and functional DEG analysis. (A) MDS; each point represents an individual sample. Yellow and purple dots indicate control (Normal) and treatment (Heat) groups. (B) DEGs volcano plot overview. log_2_ FCs in Heat-to-Normal gene expression ratios and –log_10_* p* are indicated on the *x* and *y* axes, respectively. Purple dots denote significantly 220 up- and 209 down-regulated DEGs (FDR < 0.05, absolute log_2_ FC ≥ 1). Grey dots indicate non-significant DEGs, horizontal lines mark the significance threshold (FDR < 0.05), and vertical lines establish the two FC thresholds (absolute log_2_ FC ≥ 1). KEGG pathways of the DEGs *x*-axis displays fold enrichment values; red coloured dots indicate low *p*. Each plot includes 1. Total, 2. Up-regulated and 3. Down-regulated DEGs.

RNA sequencing data processing results revealed that Normal and Heat groups averaged 34,923,354 raw reads and 50.82% GC; trimmed average reads were 34,628,356 and 50.80% GC. Alignment results for the six samples were 70.99% unique aligned reads and 92.87% overall alignment rate (Supplementary table 1). All the samples were deemed suitable to advance to the subsequent stage of the process. Multidimensional scaling (MDS) analysis compared gene expression patterns and identified distinct clusters in both groups (Fig. 1A). The DEG profiling analysis identified 429 DEGs, including 220 up-regulated and 209 down-regulated (Fig. 1B).

### Functional annotations

GO and KEGG enrichment analyses identified ambient temperature-induced functional changes (Supplementary tables 2 and 3). A treemap with a − log_10_ *p* value box size visualized GO enrichment analysis results (Supplementary figure 1), substantiating that ‘*Positive regulation of cell migration*’ was a paramount GO term in the Heat group and ‘*Cell division*’ was predominant in the Normal group. The KEGG database determined significant pathways (Fig. 1C) corroborating GO results. Protein and lipid metabolite-related terms, such as ‘*Tryptophan Metabolism*’, ‘*Metabolic Pathways*’ and ‘*Adipocytokine Signaling Pathway*’, were prevalent in the Heat group by indicating the lowest *p* value, contrasting ‘*Cell Cycle*’ and ‘*DNA Replication Pathways*’ in the Normal group shown as the lowest *p* value (Supplementary figure 3).

### GSEA

**Figure 2.**
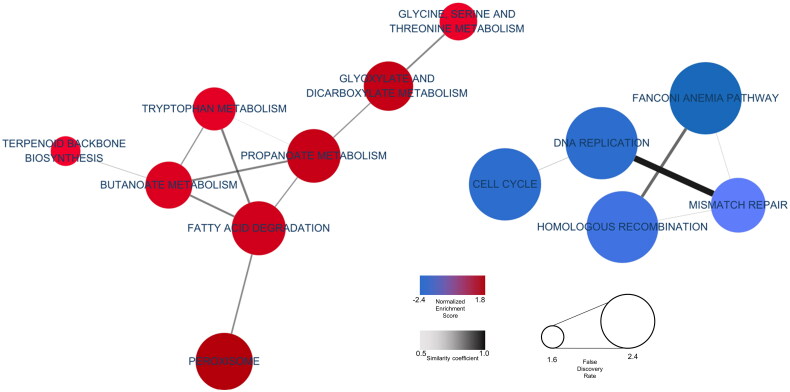
KEGG GSEA. Cytoscape visualized functional enrichment analysis results from GSEA. Nodes represent enriched KEGG pathways. Line thickness indicates overlap coefficients (overlap levels between nodes).

To assess which functional changes are affected by heat stress, we calculated enrichment score (ES) of each group an ES was adjusted for multiple hypothesis testing to confirm GSEA results which considering cumulative gene changes (Supplementary table 4). GSEA results were visualized as a cluster network based on significant terms by combining representative functions (Fig. 2). Each node’s colour signifies the NES; higher scores were expressed as thicker colours. Similarly, node size indicates the FDR; the size increases as FDR lessens. In addition, edge thickness conveys coefficient degree overlap. The network identified lipid and protein metabolism-related terms, such as ‘*Fatty Acid Degradation*’, ‘*Propanoate Metabolism*’, ‘*Butanoate Metabolism*’ and ‘*Tryptophan Metabolism*’ as highly clustered NES values by indicating NES > 1.0 and adjusted *p* value <0.005. Corroborating GO and KEGG database functional analyses, ‘*DNA Replication Pathways*’ and ‘*Cell Cycle*’ terms indicated lowly clustered NES values by indicating NES > 1.0 and adjusted *p* value <0.005. Notably, the functional analysis results derived from DEGs and the results indicating the extent of gene functional involvement, as determined by ranking all of genes, exhibited high similarity between two methods. In addition, ‘*Fatty acid degradation*’ and ‘*Tryptophan Metabolism*’ have high similarity coefficient with other pathways in the network (Fig. 2).

### Jejunal microbial classification from amplicon sequencing and diversity analysis

**Figure 3.**
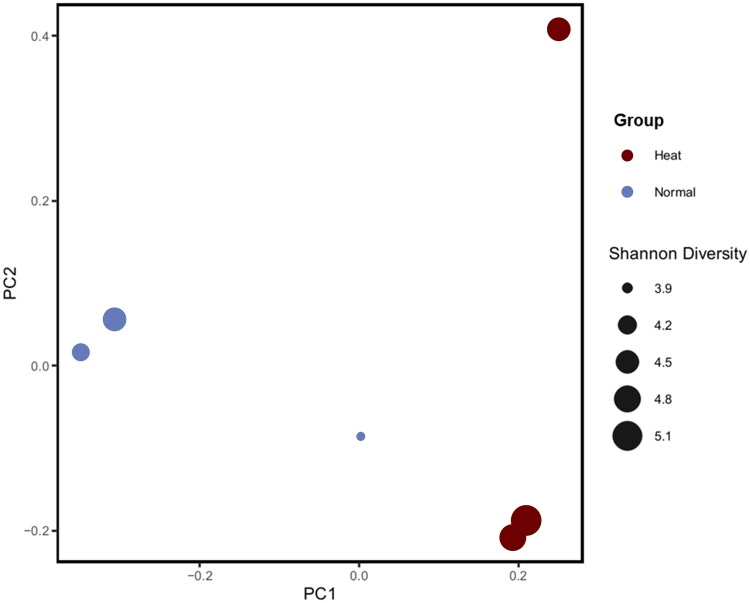
PCoA using weighted UniFrac distance, including Shannon Index information between samples. Blue indicates the Heat group, and red represents the Normal group. Node size denotes the Shannon Index.

Six jejunal samples (three Normal and three Heat) were 16S rRNA sequenced by MiSeq platform. After the quality check, approximately 9,832 ASV were revealed following denoising and clustering. The Heat group expressed a relatively higher Shannon Index than the Normal group; however, there was no statistically significant difference (Supplementary figure 2). Contrary to previous results, evenness decreased in the Heat group but indicated no statistically significant difference (Supplementary table 1). Beta diversity was calculated through an unweighted UniFrac algorithm that considered species presence, absence and phylogenic branch length. Principal coordinate analysis (PCoA) downscaled beta diversity analysis results, then alpha and beta diversity results were merged and visualized (Fig. 3). PCoA downscaled beta diversity analysis indicates that the cluster of samples by each of the group.

### Taxonomic classifications under high ambient temperature

*Firmicutes* were the dominant bacterial phylum in the Heat and Normal groups (Supplementary figure 3); however, a lower relative abundance was observed in the Heat group (90.68%) compared to the Normal group (98.56%; Supplementary table 5 and Supplementary figure 3). Next, *Bacteroidetes* were the second most abundant phylum in the Heat group (7.06%), notably elevated compared to Normal groups (0.36%; Supplementary table 5 and Supplementary figure 3). Unlike normal ambient temperature conditions, *Proteobacteria* continuously intensified under heat stress (Supplementary table 5 and Supplementary figure 3). The Heat and Normal group F/B ratio was also considerably curtailed in heat-stressed conditions.

**Figure 4.**
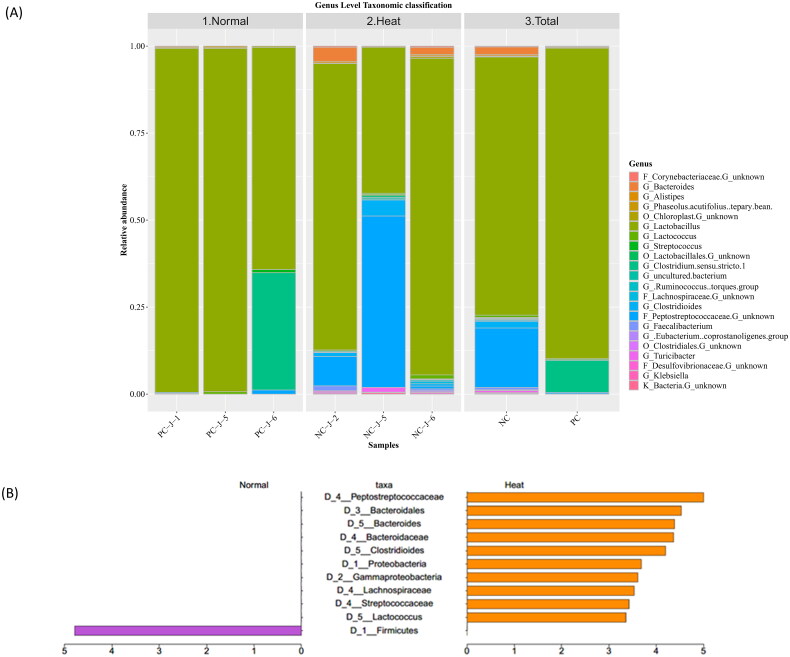
(A) Taxonomy analysis results. Colours indicate each taxon, and legends indicate taxa with more than 0.05 relative abundance. (B) Linear discriminant analysis effect size (LEfSe) results of differentially abundant taxa with an LDA threshold >2.0.

*Lactobacillus* was the dominant genus-level taxa in Heat (69.85%) and Normal groups (77.07%; Fig. 4A). Heat stress abated *Lactobacillus* abundance compared to standard ambient temperatures. *Clostridium sensu stricto 1* consistently escalated in the Heat group, whereas *Lactococcus* and *Peptostreptococcaceae* continually declined.

### Differentially abundant taxonomic analysis

Since LEfSe emphasized statistical significance, it was selected to identify specific bacterial taxa enrichment between environmental conditions. At a 2.0 LDA cut-off between groups, the phylogenic dendrogram plots include 11 differentially abundant microorganisms (Fig. 4B). Consistent with taxonomic changes in classification results, *Peptostreptococcaceae*, *Bacteroides*, *Clostridioides* and *Lactococcus* were more prevalent in the Heat group than in the Normal group (Supplementary figure 4). Comparatively, *Firmicutes* were notably depleted in heat stress conditions (Supplementary figure 4).

### Functional microbial data analysis

**Figure 5.**
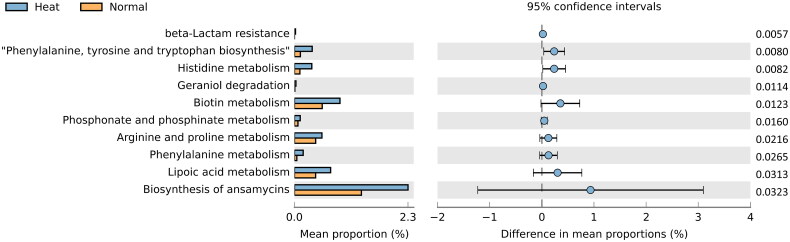
Microbial functional analysis in jejunal contents. KEGG established taxa pathways. Statistically significant pathways (*p* < 0.05) were presented by bar plot using STAMP. Orange represents the Normal group’s significant pathways; blue denotes the Heat group’s. Difference in mean proportions indicates the predicted difference in contributions for each pathway between the values.

A PICRUSt2 microbiome data analysis examined jejunum microorganisms in heat-stress conditions. Eleven statistically significant KEGG pathways were identified based on a *p* value <0.05 cut-off calculated by *t*-test (Fig. 5 and Supplementary table 6). Notably, lipid and protein metabolism-related pathways were identified as ‘*Lipoic Acid Metabolism*’, ‘*Arginine and Proline Metabolism*’, ‘*Phenylalanine, Tyrosine*’ and ‘*Tryptophan Biosynthesis*’ in Heat stress group. In addition, pathways that overlapped with transcriptome functional annotation analysis results included ‘*Lipoic Acid Metabolism*’, ‘*Phenylalanine, Tyrosine and Tryptophan Biosynthesis*’ and ‘*Histidine Metabolism*’ in Heat stress group. Due to these pathways were identified microbial functional changes in heat stress condition, these pathways were further analysed to identify microbiome–host interaction.

### Microbiome-metabolite-transcriptome integration analysis

**Figure 6.**
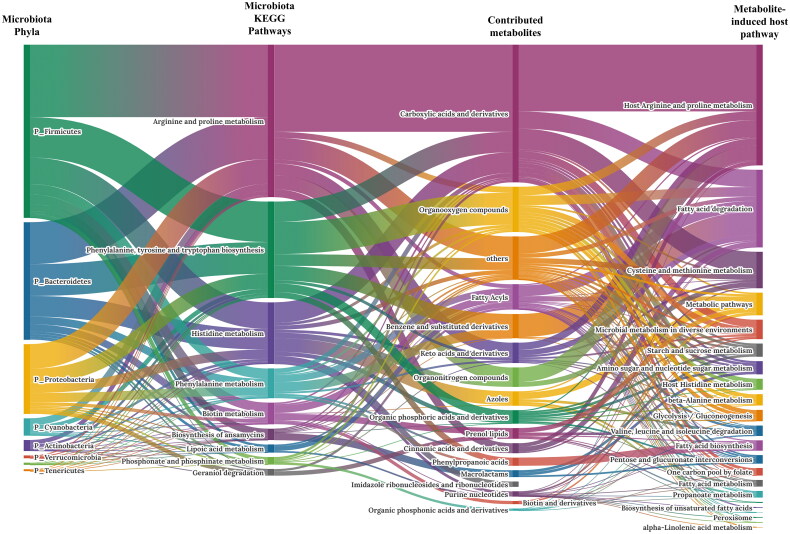
Sankey diagram of linked microbial phylum, metabolic response, inferred metabolite and host target interactions. Each taxon was represented by phylum and connected through metabolic reaction. Metabolic reactions were grouped into KEGG pathway categories. Metabolites were grouped by class in PubChem. Predicted metabolite pathways are shown in the last column.

**Figure 7.**
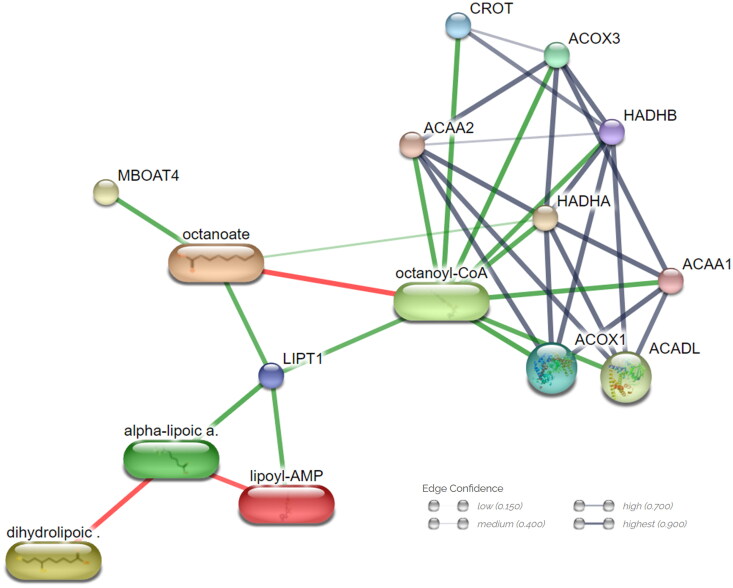
Microbiota impacts ‘*fatty acid degradation pathway*’ metabolites and gene network. Network nodes represent proteins; each node signifies all proteins produced by a single, protein-coding gene locus. Edges indicate protein–protein associations; stronger associations are illustrated with thicker lines. Protein–protein interactions are shown in grey, chemical–protein interactions in green and chemical interactions in red.

The PICRUSt2 database identified 85 microorganisms, which is contributing to the significant pathways, and the KEGG database matched 338 pathway-contributing metabolites to InChIKey from statistically significant 11 pathways and clusters with 17 categories based on class level of HMDB database. The STITCH database established 22 metabolite-induced pathways by analysing contributing metabolite and target interactions the size of the box indicates the number of contributing factors. The fatty acid degradation pathway were identified as one of largest microbiome induced metabolite contributing pathways and overlapped with GSEA results in the transcriptome analysis (Fig. 6). Furthermore, a PPI network analysis delineated metabolite host contributions in fatty acid degradation analysis. Anticipated functional partner proteins clustered with octanoyl-CoA included Acyl-CoA Oxidase 1 (*ACOX1)*, Acyl-CoA Oxidase 3 (*ACOX3*), Acetyl-CoA Acyltransferase 1 (*ACAA1*), Acetyl-CoA Acyltransferase 1 (*ACAA2*), Hydroxyacyl-CoA Dehydrogenase Trifunctional Multienzyme Complex Subunit Alpha (*HADHA*), Hydroxyacyl-CoA Dehydrogenase Trifunctional Multienzyme Complex Subunit Beta (*HADHB*) and Acyl-CoA Dehydrogenase Long Chain (*ACADL*) (Fig. 7).

**Figure 8.**
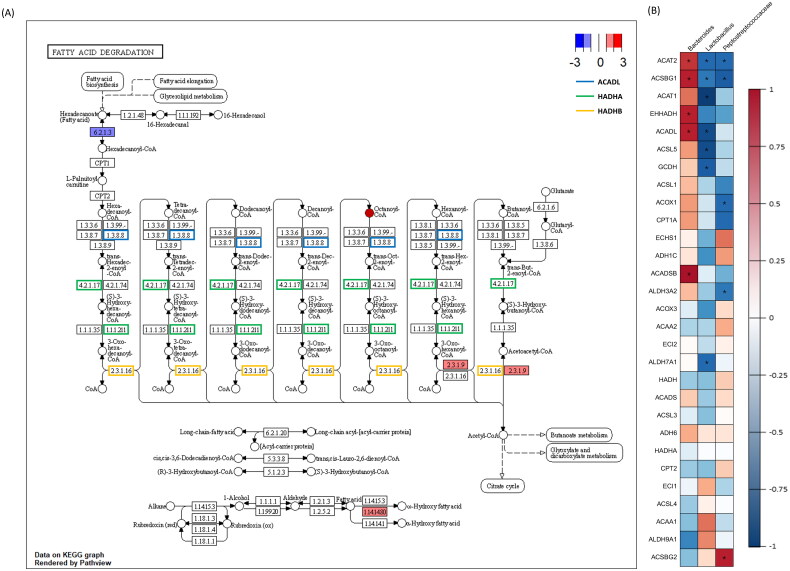
‘*Fatty acid degradation pathway*’ and associated gene and taxon correlations. (A) Red and blue rectangles represent up- and down-regulated genes. Each coloured edge indicates metabolite-induced host genes related to the fatty acid degradation pathway. Octaconyl-CoA was identified as a microbiota-induced protein. (B) Correlation plot between genes and taxa related to fatty acid degradation. The figure portrays 29 genes from this pathway and three microorganisms that passed the thresholds. * indicates *p* < 0.05 and *r*^2^ > 0.7.

Additionally, pathway-based integration analysis of fatty acid degradation pathways with host DEGs predicted functional partner proteins and metabolites from microbiota were assessed (Fig. 8A). Differentially expressed host genes contributed to the fatty acid degradation pathway’s beginning and end. Other predicted metabolites and functional partner proteins were primarily involved at the pathway’s midpoint.

Correlation analysis evaluated 22 fatty acid degradation pathway-contributing genes and three microorganisms (*Bacteroides*, *Lactobacillus*, *Peptostreptococcaceae*). *Bacteroides* exhibited a positive (*r*^2^ > 0.7) association with Acetyl-CoA Acetyltransferase 2 (*ACAT2)*, Acyl-CoA Synthetase Bubblegum Family Member 1 (*ACSBG1*), Enoyl-CoA Hydratase And 3-Hydroxyacyl CoA Dehydrogenase (*EHHADH*), Acyl-CoA Dehydrogenase Long Chain (*ACADL*) and Acyl-CoA Dehydrogenase Short/Branched Chain (*ACADSB*), whereas *Lactobacillus* negatively correlated with *ACAT1*, *ACADL*, Achaete-Scute Family BHLH Transcription Factor 5 (*ASCL5*), Glutaryl-CoA Dehydrogenase (*GCDH*) and Aldehyde Dehydrogenase 7 Family Member A1 (*ALDH7A1*). *Peptostreptococcaceae* negatively correlated with *ACAT2*, *ACSBG1*, *ACOX1* and carnitine palmitoyltransferase 1 A (*CPT1A*) and positively correlated with Acyl-CoA Synthetase Bubblegum Family Member 2 (*ACSBG2*).

## Discussion

### Functional DEGs among intestinal tissues under heat stress

Functional annotation analysis results of host using the KEGG database revealed ‘*Tryptophan Metabolism*’ and ‘*Glycine, Serine and Threonine Metabolism*’ as significant pathways in the functional annotations of up-regulated DEGs (Fig. 1D). Consistently, the GSEA results confirmed that the ‘*Protein Metabolism*’ and ‘*Fatty Acid Degradation Pathways*’ were significant. Tryptophan, glycine and threonine compositional changes occur in broilers under heat stress for energy supply.^41^ Including tryptophan and glycine, the jejunum digests and delivers nutrients to the body.^42^ Our results suggest that changes in mRNA expression on the surface of the jejunum lead to distortions in the digestion and absorption of amino acids. In addition, broiler chickens regularly use adipocytokines for energy metabolism when exposed to heat stress. In other livestock breeds, including other broilers, adipocytokine signalling contributes to stress adaptation by activating energy metabolism in chronic high-temperature scenarios.^41^^,^^43^^,^^44^

Fatty acid degradation identified in GSEA results includes oxidation (Fig. 2), and chronic heat stress during lipid peroxidation prompts cytochrome C reduction and the production of hydroxyl radicals.^45^ Moreover, oxidative lipid degradation promotes cell death by producing peroxides^46^; the subsequent cell reproduction regulation inhibits nutrient digestion and body weight gain. Indeed, statistically significant decreases in growth performance such as body weight, bodyweight gain and feed intake were observed in the same experiments.^27^ Furthermore, strengthened fatty acid degradation in the jejunum, which is responsible for digestion and absorption, contributes to the adaptation process by increasing energy metabolism during heat stress. A similar study on other animals confirmed that appetites waned when elevated fatty acid oxidation augmented energy metabolism.^47^ Therefore, the reduced appetite in broilers due to heat stress was likely caused by increased fatty acid decomposition in the jejunum.

Alternatively, the KEGG pathway analysis and GSEA using down-regulated DEGs revealed involvement of cell cycle and DNA replication pathways (Figs. 1E and 2). Jejunal tissues produce heightened shock proteins when exposed to high ambient temperatures.^48^ Denatured and aggregated proteins damage intestinal cells and mucosa,^49–51^ diminishing feed intake, feed efficiency and nutrient transporters that aid luminal bacteria invasion.^52^ Moreover, heat stress leads to apoptosis of intestinal epithelial cells as a result of elevated reactive oxygen species.^53^ Our analysis of differentially expressed genes (DEGs) shows up-regulation of functional pathways related to fatty acid oxidation, suggesting that cell cycle disruption, DNA replication and fatty acid degradation may impede the recovery of damaged jejunal tissue.

### Taxonomic modification under ambient temperature modification

According to the taxonomic analysis of microorganism classification, the *Firmicutes* phylum and *Lactobacillus* genus were dominant in both groups, but they were markedly higher in the Heat group (Fig. 4).^54^^,^^55^ Our findings support previous reports that Firmicutes and Lactobacillus are the predominant species in the jejunal microbiota of swine.^56^ Notably, previous studies have suggested that a low abundance of Lactobacillus increases intestinal permeability and disrupts obesity metabolism, which aligns with the observations in our study. Thus, the *Firmicutes/Bacteroidetes (F/B)* ratio correlated with host–microbiota imbalance and obesity degree, significantly prevalent in the heat-stressed group.^57^

The Heat group exhibited remarkably abundant *Peptostreptococcaceae* and *Bacteroides,* while the Normal group showed a strikingly high abundance of *Firmicutes* and the *Lactobacillus* genus (Fig. 4). Elevated levels of the Bacteroides genus in the intestines led to intestinal dysbiosis, which dominated the degradation efficacy in the gut and altered fatty acid metabolism.^58^ Previous studies have observed that dysbiosis, characterized by low Lactobacillus levels of Lactobacillus, can lead to increased intestinal permeability and contribute to metabolic dysfunction associated with obesity, which is consistent with our findings.^59^

A microorganism’s metabolism produces short-chain fatty acid (SCFA) and branched-chain fatty acid as final fat and protein metabolites.^60^^,^^61^ Our findings suggest that environmental changes alter the composition of the microbiome, leading to an increase in SCFA production under heat stress as a result of increased protein and fat metabolisms (Fig. 5). SCFAs participate in host fat metabolism and increase lipid peroxidation by moulding an environment that exposes intestinal epithelial cells to lipid peroxide products.^62^ In addition, our transcriptome analysis revealed that apoptosis, proliferation and growth cessation disrupt the cell cycle when exposed to products undergoing lipid peroxidation.^63^

Moreover, lipid peroxidation products loosen tight junctions in intestinal epithelial tissue.^64^ The current study identified a term related to the synthesis of L-tryptophan induced by heat stress. Metabolic products of bacterial L-tryptophan directly influence the host’s immune system^65^ and activate epithelial barrier functions by enhancing tight junctions in pig intestinal epithelial cells.^66^ Our transcriptome analysis revealed a significant increase in tryptophan metabolism, suggesting that intestinal epithelial cells activate tryptophan in response to heat stress damage.

This study also ascertained that heat stress fluctuated microbial flora in pigs. These results indicate that the microbiome compositions showed increased abundance of the *Firmicutes* phylum and *Lactobacillus* genus in both groups, with this dominance being more pronounced in the heat-stressed group. However, the groups showed no statistically significant differences in alpha-diversity index. Heightened levels of *Peptostreptococcaceae* and *Bacteroides* in the Heat group altered fatty acid metabolism. Similarly, increased heat stress led to higher production of SCFAs, which in turn was linked to increased protein and fat metabolisms, leading to lipid peroxidation and intestinal epithelial cell disruption.

### Functional host and microbiota interactions

We noted that microbiota adapted to environmental heat stress conditions by accelerating energy metabolism (Fig. 6).^67^ Pathways related to metabolite identification indicated that the microorganism’s metabolism produced an excessive amount of metabolites for host energy production. In addition, the host contributing pathways identified from the predicted metabolites were confirmed to be primarily protein, proline and fat metabolism, including arginine and fatty acid degradation. Furthermore, the degradation products of fatty acids were found to SCFA, which influences host lipid peroxidation and cell cycle disruption.^68^ These results propose that microbiota and host transcriptomes utilize the host’s metabolites as a mediator in the fatty acid degradation process.

Clustered protein and metabolite PPI networks involved in fatty acid degradation include *ACOX1*, *ACOX2*, *ACOX3*, *ACAA1*, *ACAA2*, *HADHA*, *HADHB*, *CROT* and octanoyl-CoA (Fig. 7). By utilizing proteins, metabolites and DEGs obtained from transcriptome analyses, we confirmed that the transcripts involved in fatty acid degradation are present in both the initial and final stages. Additionally, microorganism-derived metabolites and proteins were found to contribute to the repetitive degradation process. These findings not only confirm the involvement of the transcript in the degradation process but also suggest that modifications by microorganisms influence energy metabolism, particularly affecting host fatty acid degradation.

*Bacteroides*, *Lactobacillus* and *Peptostreptococcaceae* were prevalent microorganisms involved in these interactions and exhibited differential abundance. Correlation analysis between corresponding microorganisms and genes involved in fatty acid degradation identified *ACAT2*, *ACSBG1*, *ACAT1*, *EHHADAH* and *ACADL* as highly related to three microbiota (Fig. 8). These genes encode proteins that specifically influence the stabilization induced by lipids, particularly associated with mitigating lipotoxicity through lipid-induced stabilization.^69^^,^^70^ When considered alongside changes in the microbial community, these alterations corresponded to an increase in *Bacteroides* and a decrease in *Lactobacillus* and *Peptostreptococcaceae* during heat stress. This led to modifications in key genes, including those in the *ACAT* gene family, which affect competitive fat oxidation in broiler chickens. The *ACADL* gene has a significant function in the fatty acid degradation pathway and is highly correlated with significant taxa. It catalyses the initial stage of mitochondrial fatty acid oxidation and has adverse effects on muscle and fat synthesis.^71^^,^^72^ The findings from previous study are consistent with a verified decrease in cell cycle and DNA replication, as indicated by RNA sequencing results. Thus, changes in the expression level of the *ACADL* gene, associated with the increase in *Bacteroides* and the decrease in *Lactobacillus* and *Peptostreptococcaceae*, not only affect the fatty acid degradation and oxidation but are also closely linked to cell regeneration and division within the host’s jejunum. Consequently, our results suggest that *ACADL* serves as a key gene and a potential biomarker for damage caused by heat stress. Additionally, *Bacteroides* positively correlated with *ACADSB*, which encodes the short/branched-chain acyl-CoA dehydrogenase that catalyses the dehydrogenation of acyl-CoA derivatives in fatty acid metabolisms.^73^ Since *Bacteroides* contribute to lipid metabolism and decrease the circulation of lipid peroxidation products, we hypothesize that elevated *ACADSB* gene and *Bacteroides* concentrations contribute to lipid degradation by up-regulating expression levels of other genes.

This study unveiled interactions between microorganisms and hosts mediated by metabolites to the lipid peroxidation under heat stress. We provided candidate biomarkers in microbiota and genes under heat stress conditions including *Bacteroides*, *Lactobacillus*, *Peptostreptococcaceae*, *ACADL*, *ACAT* gene family and *ACOX* gene family which are related with fatty acid degradation pathway. Our findings contribute to a more comprehensive understanding of the physiological and molecular mechanisms underlying broiler adaptations to heat stress conditions, aiding heat-stress countermeasure developments in poultry industry. However, additional metabolomic analysis, which considers quantitative data on metabolites produced by microorganisms is necessary for a more comprehensive understanding.

## Supplementary Material

Supplemental Material

Supplemental Material

Supplemental Material

Supplemental Material

Supplemental Material

Supplemental Material

## Data Availability

The datasets generated and analysed during the current study are available in the NCBI Sequence Read Archive (SRA) repository, https://www.ncbi.nlm.nih.gov/sra/PRJNA984346.
